# 509 Cytokine Profile Following Lipopolysaccharide Stimulation of Adipose-Derived Stem Cells from Burn Patients

**DOI:** 10.1093/jbcr/iraf019.138

**Published:** 2025-04-01

**Authors:** Gina DeFelice, Abdul-Razak Masoud, Paige Deville, Dhanushka Vitharana, Ada Ozcan, Kaitlyn Andre, Jeffrey Carter, Herbert Phelan, Jonathan Schoen, Victoria Miles, Alison Smith

**Affiliations:** Burn Center at University Medical Center; Louisiana State University Health Sciences Center - New Orleans; Louisiana State University Health Sciences Center - New Orleans; Louisiana State University Health Sciences Center - New Orleans; Louisiana State University Health Sciences Center - New Orleans; Louisiana State University Health Sciences Center - New Orleans; Burn Center at University Medical Center; Burn Center at University Medical Center; Louisiana State University Health Sciences Center - New Orleans; Burn Center at University Medical Center; Louisiana State University Health Sciences Center - New Orleans

## Abstract

**Introduction:**

Adipose tissue and adipose-derived stem cells (ADSCs) have been demonstrated to have an important role in the mediation of inflammatory response to injury and illness. The secretion of paracrine factors by stem cells, which can have pro- or anti-inflammatory effects, may have wider implications on wound healing. Lipopolysaccharide (LPS) is an important component of the outer membrane of gram-negative bacteria that triggers a strong immune response. The objective of this study was to compare the paracrine factors secreted from damaged adipose tissue from burn patients with and without LPS administration.

**Methods:**

Fat samples were collected from 28 burn adult patients with TBSA from 10-95% who presented to an ABA Verified Burn Center. Fluorescence-activated single cell sorting (FACS) confirmed the presence of ADSCs. Lipopolysaccharide (LPS) was administered to 12 matched burn samples to mimic an initial inflammatory response, and supernatant was collected from cell culture for analysis. The secretion of 10 paracrine factors were measured by ELISA. A Mann-Whitney u test was performed.

**Results:**

Levels of all 10 paracrine factors analyzed were greater in samples of ADSCs from burn patients with LPS administration. There was a significant difference in the levels of four specific paracrine factors: IL-6, IL-1-beta, IL-8, and IL-10 (p < 0.05).

**Conclusions:**

Our findings demonstrate increased secretion of four specific paracrine factors from ADSCs in burn patients following LPS administration. These factors are both pro- and anti-inflammatory and have been previously found to attenuate autoinflammatory syndromes. Future directions will be focused on investigating the effects of increased IL-6, IL-1-beta, IL-8, and IL-10 on wound healing in burn patients with concomitant infections.

**Applicability of Research to Practice:**

The identification of specific paracrine factors increased in burn patients, especially in those with concomitant infections, will help further understand the mechanisms of wound healing and clinical interventions to improve wound healing and mitigate infections in burn patients.

**Funding for the Study:**

N/A

